# Preventing Silica Scale Formation Using Hydroxide Ions Generated by Water Electrolysis

**DOI:** 10.3390/membranes9110154

**Published:** 2019-11-15

**Authors:** Yoshihiko Sano, Masataka Yamaguchi

**Affiliations:** Department of Mechanical Engineering, Shizuoka University, 3-5-1 Johoku, Naka-ku, Hamamatsu 432-8561, Japan; fizzwater92@gmail.com

**Keywords:** preventing silica scale, water electrolysis, polymerization reactions, ion exchange membrane

## Abstract

The reaction of silica with various cations in a solution and with hydroxide ions generated by water electrolysis was investigated as a means of preventing the formation of silica scales in geothermal binary power generation. Through batch and continuous experiments, it was found that all silica in the cathode phase of a reaction device could be removed if the necessary amounts of magnesium and calcium were present. This occurs because a silica-magnesium-calcium compound is produced via a polymerization reaction with cations in a solution and with hydroxide ions generated by electrolysis. Analysis by inductively coupled plasma and energy dispersive X-ray spectroscopy shows that this material has the formula 2CaO-5MgO-8SiO_2_-H_2_O, and thus is likely generated by the reaction proposed by Sheikholeslami et al. (2019). Increasing the current sent through the reaction solution subsequently produces calcium carbonate. This technique for the separation of silica and calcium from aqueous solutions can be operated continuously without channel clogging, which indicates the possibility of practical applications. However, overly high currents promote the migration of protons from the anode to cathode phases, which inhibits the formation of precipitates due to a neutralization reaction. The proposed method is an effective approach for removing silica from a solution in geothermal binary power generation; although, a means of suppressing the effects of proton generation will be necessary if the process is also to be used to remove calcium ions.

## 1. Introduction

Geothermal energy has been utilized to generate electricity and to provide heating, and it represents a stable energy supply that reduces greenhouse gas emissions [1]. One drawback associated with this energy source is that the minerals in geothermal brine tend to form deposits known as scale when their solubility limit is exceeded, which decreases the energy recovery efficiency from geothermal resources. This scale typically consists of calcium carbonate and silica [2], and is an issue not only in geothermal plants [3,4], but also in reverse osmosis systems [5] and cooling towers [6]. There have been many studies on the prevention and removal of scale. Calcium carbonate scale is formed by the reaction between calcium and carbonate ions. Polyacrylic acid [7,8] and hydrochloric acid [9] can be used to reduce such scaling, but are costly and are not environmentally friendly. Recently, Sano and Nakashima [10] suggested that calcium carbonate scale could be prevented by adding directly electrolyzed water into geothermal brine. This method is inexpensive and has a low environmental burden, but the extent to which it prevents the formation of silica scale has not been assessed.

It is well known that silica scale is harder than calcium carbonate scale, and so is more difficult to remove from the surfaces of heat exchangers and pipes. The equilibrium concentration of dissolved silica depends on the temperature, pH and chloride ion concentration in a solution [11], and silica scale is sometimes diminished by avoiding supersaturation or by adding chemical inhibitors [12,13]. Al(OH)_3_ [14], magnesium salt [15,16], silica gel seeds [17] and other chemicals [18,19] have also been used to induce the precipitation of silica. In addition, Gabelich et al. [20] reported that increasing the pH of an ionic solution effectively induces the precipitation of silica, with significant removal of silica above a pH of 10. Sheikholeslami et al. [21] and Nicholas et al. [22] determined that silica could be removed via the following precipitation reactions:

Mg(HCO_3_)_2_ + 3H_4_SiO_4_ → MgSi_3_O_6_(OH)_2_ + 6H_2_O + 2CO_2_ (1)

Ca^2+^ + H_4_SiO_4_ + 2OH^−^ → CaSiO_3_ + 3H_2_O (2)

2Ca^2+^ + 5Mg^2+^ + 8H_2_SiO_3_ + 14OH^−^ → 2CaO-5MgO-8SiO_2_-H_2_O + 14H_2_O. (3)

Electrocoagulation can also be used to remove silica that occurs as a result of reactions with the polyvalent metal cations and the hydroxide ions generated by sending an electric current through a solution via two parallel aluminum or iron plates [23,24,25]. This process is believed to involve reactions similar to those shown in Equations (1)–(3). According to Nicholas et al. [22], electrocoagulation using a sacrificial anode is advantageous because it allows remote operation with compact equipment and remote control compared to chemical coagulants. However, residual aluminum may remain in the solution after this process. Furthermore, the sacrificial anode is consumed by the electrode reaction during this process, and so must be periodically replaced. On the other hand, electrodeposition, which has long been exploited to modify electrode surfaces [26], relates to these electrocoagulation techniques, and silica thin films have been synthesized on an electrode by electrodeposition [27,28,29]. Tuning the pH value at the electrode/solution interface region is of importance for the synthesis of metal oxides and hydroxides in the cathodic electrodeposition [27,30,31], indicating that the hydroxide ion is a key for removing silica and metal from aqueous solutions.

Recently, Sano et al. [32] proposed a similar precipitation technique for the recovery of magnesium resources from seawater. This process is based on reactions with cations in a solution and with hydroxide ions generated by the electrolysis of water in a cathode channel separated by an ion exchange membrane. Using this method, 99% pure magnesium hydroxide was obtained from seawater. This result suggests the possibility that silica can be removed using the same technique, without the dissolution of polyvalent cations from a sacrificial anode. Moreover, Equation (2) indicates that calcium ions would also be removed from the solution, meaning that calcium carbonate scale would also be prevented.

The present study evaluated the reactions of silica species with cations in a solution and hydroxide ions generated by water electrolysis as a means of preventing the formation of calcium carbonate and silica scales in geothermal binary power generation. Normally, though geothermal binary power generation does not require the removal of all the silica contained in the groundwater, it is required to reduce the silica concentration with a simple system that has little environmental impact. In this study, hot spring water obtained from Atagawa and Katase, Japan, both of which are candidate sites for geothermal binary power generation, was used as the experimental solutions. In the system employed in this work, a platinized titanium plate was used as the electrode instead of a sacrificial anode. During batch trials, hot spring water underwent electrolysis in a test chamber separated by a cation ion exchange membrane to assess the resulting reactions. The precipitates obtained were subsequently analyzed by scanning electron microscopy (SEM) and energy dispersive X-ray spectroscopy (EDS). In addition, ion concentrations in the various solutions were determined using inductively coupled plasma atomic emission spectroscopy (ICP-AES) to provide further information with regards to the various reactions during the proposed method. Following these trials, continuous process experiments were carried out under flow conditions to assess the feasibility of practical industrial applications. 

## 2. Experimental Methods

**Figure 1:**
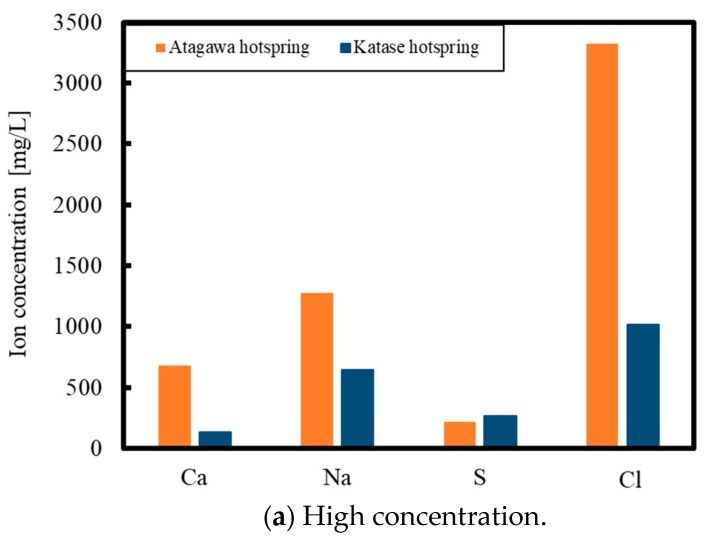

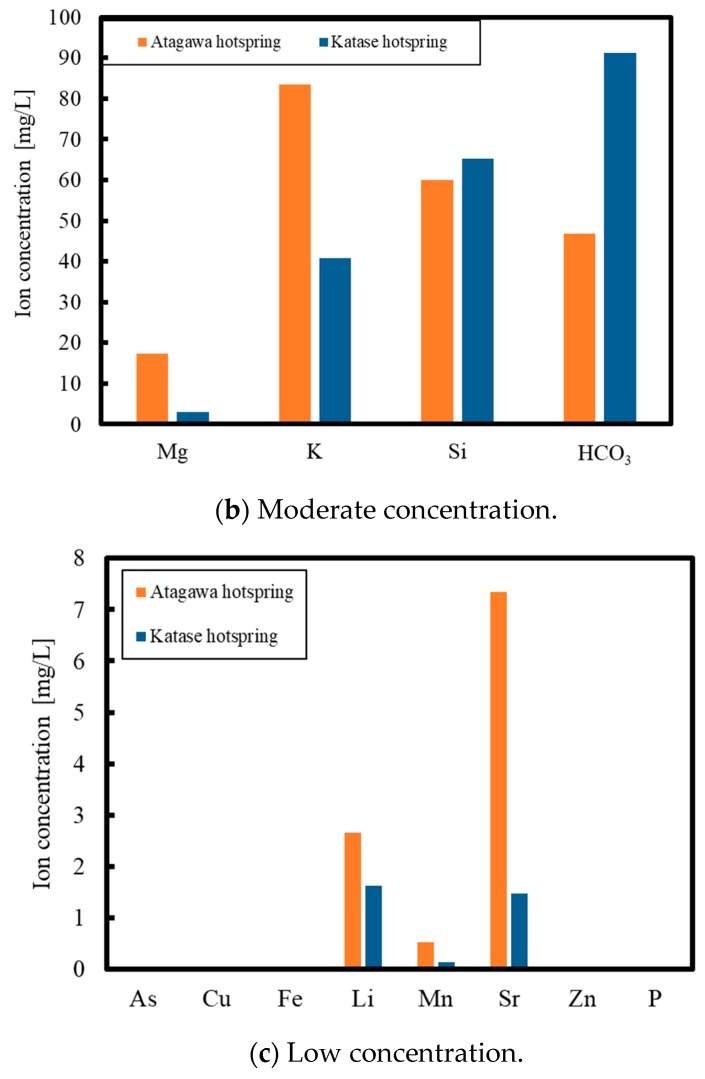
Elemental concentrations in hot spring water obtained from Atagawa and Katase, Japan. (**a**) High concentration; (**b**) Moderate concentration; (**c**) Low concentration.

As noted, hot spring water obtained from Atagawa and Katase, Japan, was used in the experiments. Figure 1 summarizes the elemental concentrations in each sample as determined by ICP-AES (Optima8300, PerkinElmer, Massachusetts, USA), high-performance liquid chromatography (HPLC, SPD-20A, CDD-10Avp/10Asp, Shimadzu, for bicarbonate ions) and ion analysis (IA-300, DKK-TOA Co., for chloride ions, Tokyo, Japan). These data show that numerous elements other than calcium and silica are present in the hot spring water. The calcium ion concentration is also much higher than the silica concentration, indicating the possibility that all silica could be removed by the reactions shown in Equations (2) and (3). While there are differences in the ion concentrations between the two water samples, both have essentially the same level of silica. For this reason, either type of water could be used for the initial trials, and the Atagawa specimen was selected for the batch experiments, while the Katase sample was employed during the continuous experiments. 

**Figure 2:**
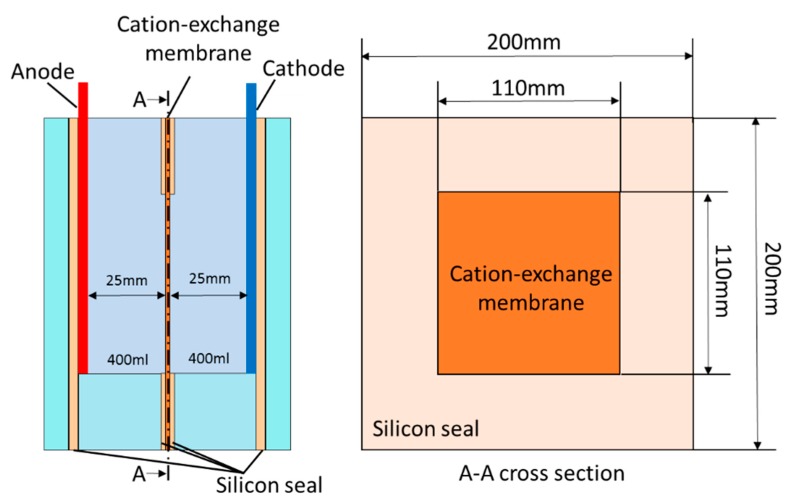
Experimental equipment used in batch process trials.

Figure 2 presents the experimental apparatus used during the batch trials. This equipment consisted of an acrylic plate, silicone rubber sheets, platinum-plated electrodes and a divalent cation exchange membrane. The silicone rubber acted to prevent water leakage and to hold the membrane (CMB, ASTOM, Tokyo, Japan) in place. As shown in the figure, the membrane was placed at the center of the experimental device and sandwiched between two silicone rubber seals, providing an exposed area with dimensions of 110 mm × 110 mm through which ions could pass. Platinum-plated electrodes (DENBOH, Gunma, Japan) were placed at each end of the cell, 25 mm from the ion exchange membrane. Table 1 provides the manufacturer’s specifications for the cation exchange membrane. 

In the batch experiment, 400 mL samples of the Atagawa water were filtered (240 mm, 5C, pore size 1 µm, Advantec, Tokyo, Japan) and then transferred into the anode and cathode tanks. The electrolysis trials were performed under constant current conditions (0.8, 1.6 or 2.4 A), using a DC power supply (GEO142938, GWinstek, Tokyo, Japan), for durations of 1, 2, 5 or 30 min, or for 1, 5, 12 or 24 h. Following each experiment, aliquots were removed from both the anode and cathode tanks and transferred into beakers, and then left for 30 min so as to allow any chemical reactions to proceed to completion. The pH values for the samples were measured using a pH meter (MM60-R, DKK-Toa Corp., Tokyo, Japan). These aliquots were subsequently filtered (240 mm, 5C, pore size 1 µm, Advantec) to separate the precipitate from the aqueous solution. The precipitate was then dried in an oven (OFW-450B, ASONE Tokyo, Japan), after which surface observations and structural analyses were conducted using SEM (TM3030Plus, Hitachi, Tokyo, Japan) and EDS (TM3000 MICSF+, TM3000 XSTREAM2, Oxford Instruments, Oxfordshire, UK). The elemental composition of the precipitate was determined by searching for all of the elements from beryllium to uranium. The elemental concentrations in the separated aqueous solution were determined using ICP-AES (Optima8300, PerkinElmer).

**Figure 3:**
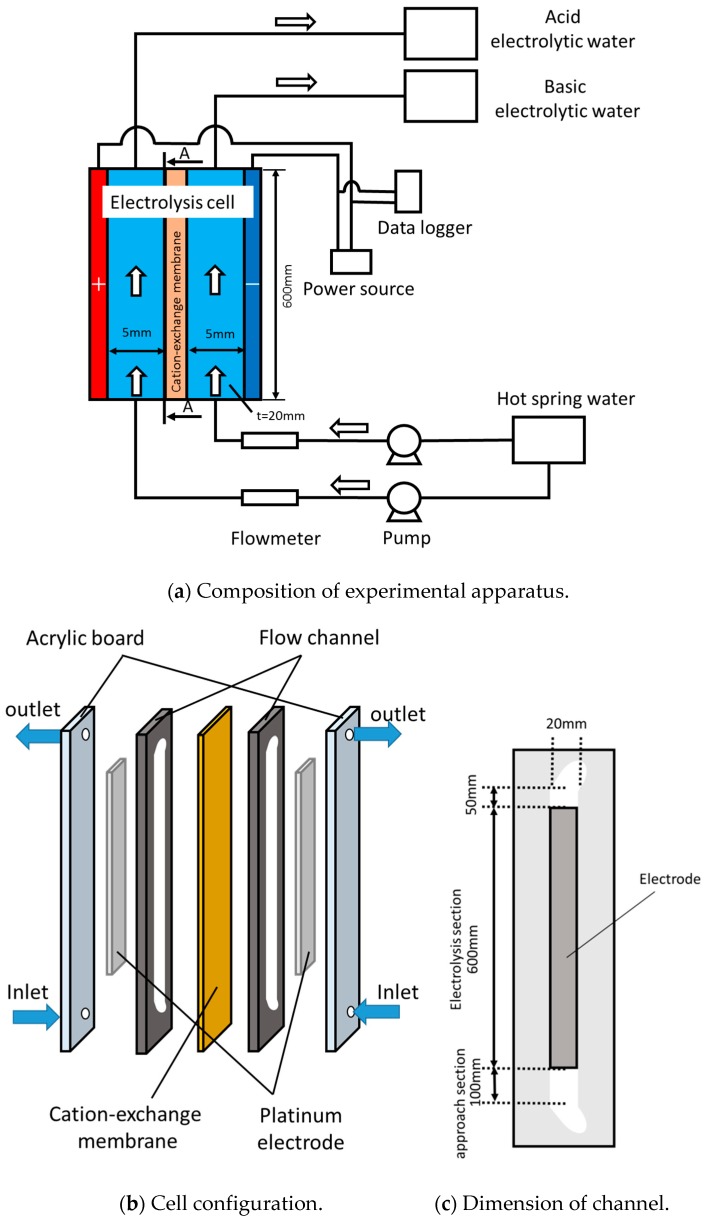
Experimental apparatus used during continuous process trials. (**a**) Composition of experimental apparatus; (**b**) Cell configuration; (**c**) Dimension of channel.

The flow type electrolytic device employed during the continuous process trials is illustrated in Figure 3a. This device consisted of an electrolytic cell, pump, flow meter, drain tank, hot spring water tank, voltmeter and power source. Hot spring water obtained from Katase was provided to the anode and cathode channels in the electrolytic cell using two pumps (MG204XPD17-10S, Magpon Gear, Tokyo, Japan). The flow rates were controlled by inverters (FY-S1NO08S, Panasonic, Osaka, Japan) connected to each pump, and monitored using float type flowmeters (GL200A, Graphtec). The current was provided to the platinum-plated electrodes (600 mm × 20 mm × 5 mm, Denboh, Gunma, Japan) and was installed at the ends of each channel by a DC power supply (GEO142938, GW instek, Yokohama, Japan) to occur water electrolysis in the flowing solution. The current was recorded by a data logger (GL200A). Samples were taken at the outlet of each channel under steady state conditions. The assessments of the elemental concentrations in the solution, as well as surface observations and structural analyses of the precipitate, were performed using the same processes as described above with regard to the batch tests.

The structure of the electrolysis cell is shown in Figure 3b. The unit consisted of an acrylic plate, platinum-plated electrodes, a cation exchange membrane and silicone rubber sheets, and was designed for continuous processing but was based on the batch device, such that the same materials were used. The flow channels were created by the rubber sheets, which also served as gaskets. The ion exchange membrane was sandwiched between the rubber sheets and was positioned at the center of the electrolytic cell, so that two channels were formed, representing the anode and cathode. The platinum-plated electrodes were placed in grooves in the acrylic plate so that the flow channel walls were smooth. Figure 3c provides the dimensions of the electrolytic channel. The channel had a length of 750 mm, a width of 20 mm and a depth of 5 mm, while the electrodes had a length of 600 mm and a width of 20 mm. Therefore, the effective membrane area was equal to the electrode area (that is, 600 mm × 20 mm). The additional sections with lengths of 100 mm and 50 mm before and after the test section, respectively, were provided to prevent flow deviation.

In each batch experiment, hot spring water obtained in Katase was first filtered (240 mm, 5C, pore size 1µm, Advantec) and then used as the test solution. The water was supplied to the anode and cathode channels at a flow rate of 50 mL/min, flowing against gravity, and the electrolysis was performed under constant current conditions, with currents between 0.2 and 5.0 A. As was also reported by Sano et al. [32], clogging of the flow channels did not occur as a result of employing a flow direction against gravity.

## 3. Results and Discussion

### 3.1. Batch Process

**Figure 4:**
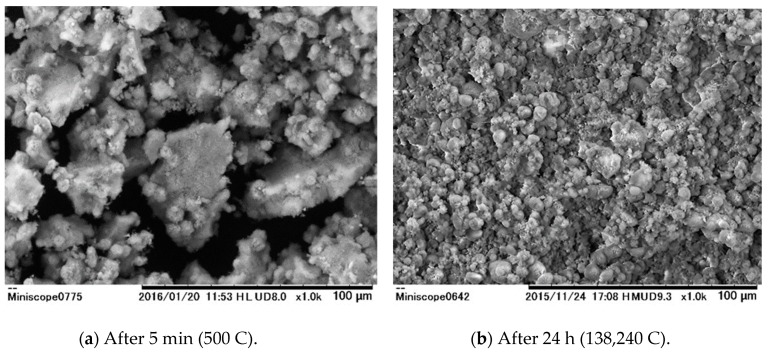
Scanning electron microscopy (SEM) images of precipitates obtained from batch trials under various conditions. (**a**) After 5 min (500 C); (**b**) After 24 h (138,240 C).

The formation of a white precipitate was only observed in the cathode phase when the hot spring water was electrolyzed, and this precipitate was found to adhere to the cathode plate. This material was found to peel away from the plate when the current direction was reversed. In this study, the electrode was cleaned up by hydrochloric acid before the experiment, and was used in all experiments. Figure 4a,b show SEM images of the solids obtained from the cathode phase in batch experiments with durations of 5 min and 24 h at 1.6 A, respectively. It is evident that, after 5 min of electrolysis, small particles (10 μm or less) are deposited on the surfaces of relatively large particles (50 μm). In contrast, after 24 h of electrolysis, the small particles are more numerous and fill the image. These results indicate that different types of precipitates are obtained depending on the electrolysis time.

Table 2 summarizes the results of the quantitative analyses of both solids shown in Figure 4a,b. Note that carbon is not included, since a carbon plate was used during the analysis process. After 5 min of electrolysis, the magnesium, silica, calcium and oxygen molar ratio was 1:1.25:1:7, while the ratio was 1:1:20:57 after 24 h of electrolysis. These data suggest that the relatively large deposits shown in Figure 4 are composed primarily of magnesium, calcium and silica, while the smaller formations are largely made of calcium.

**Figure 5:**
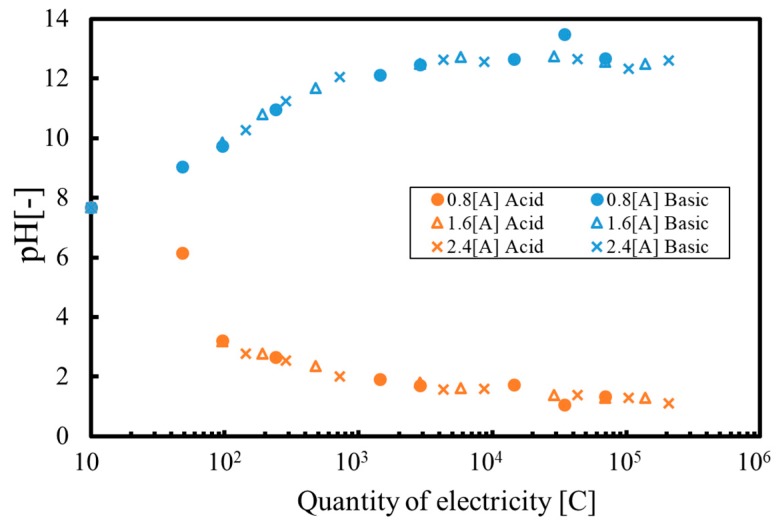
pH values during batch tests as functions of quantity of electricity.

Figure 5 plots the pH values against the quantity of electricity for several different conditions. It can be seen that the pH values follow a single trend line in each plot, indicating that the chemical reactions associated with precipitate formation are dependent on the quantity of electricity. Furthermore, variations in the pH value in the cathode phase had less of an effect than that in the anode phase, which demonstrates that hydroxide ions generated in the reaction solution were used to create the precipitate at the cathode side. Furthermore, the data show that the pH values tend to plateau when employing high coulomb values. The quantity of each ion passing through the ion exchange membrane is proportional to the molar ratio of the ion in a solution. The proton concentration in the anode tank increases with electrolysis time, so that protons migrate from the anode phase to the cathode phase in the case of a high amount of electricity. Simultaneously, neutralization reactions occur to produce an equilibrium pH value.

**Figure 6:**
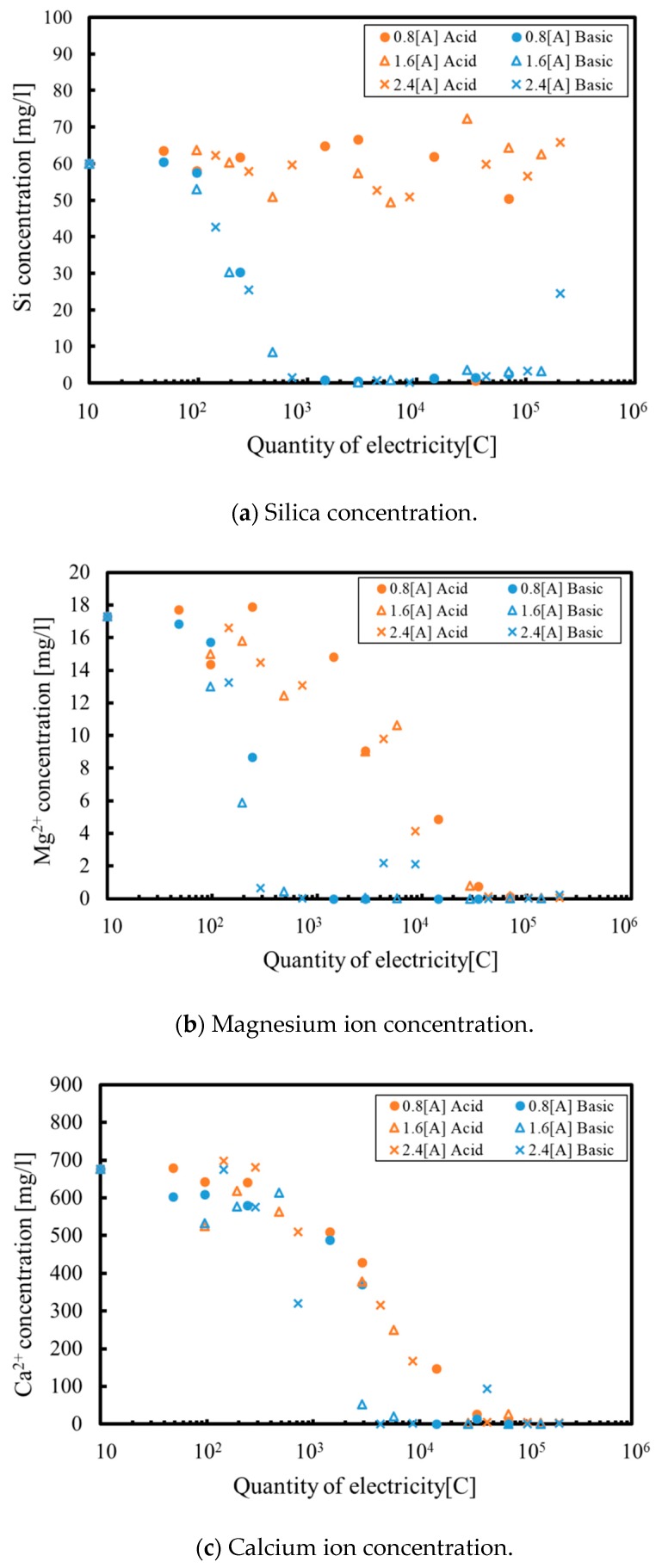

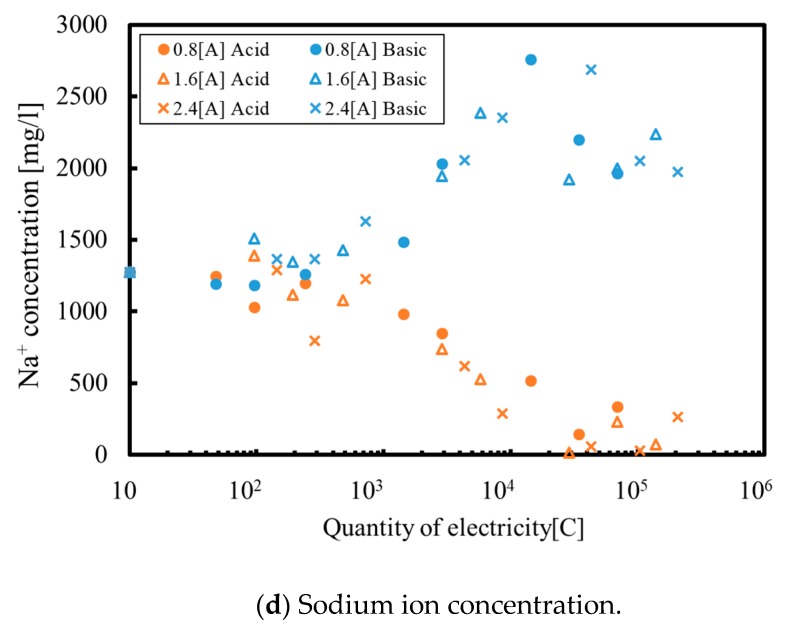
Elemental concentrations obtained by an inductively coupled plasma atomic emission spectroscopy (ICP-AES) as functions of the quantity of electricity under various reaction conditions. (**a**) Silica concentration; (**b**) Magnesium ion concentration; (**c**) Calcium ion concentration; (**d**) Sodium ion concentration.

Figure 6 plots the concentration of various elements in the electrolyzed solution, as determined by ICP-AES. Figure 6a demonstrates that the silica concentration in the cathode phase decreases as the quantity of electricity increases when the hot spring water undergoes electrolysis via the proposed method. Furthermore, we can confirm that silica is completely removed from the solution when 10^3^ C has been applied. Conversely, the silica concentration in the anode phase is relatively constant because the silica is not positively charged, and thus, cannot pass through the ion exchange membrane.

In contrast, as can be seen in Figure 6b, the magnesium ion concentration decreases in the anode phase, since magnesium ions are positively charged. Magnesium is removed from the solution in the cathode phase because chemical reactions occur to generate a precipitate in this phase. As a consequence, magnesium can be removed from both the anode and cathode phases, and is completely removed from all solutions at 30,000 C. Calcium exhibits the same trend as the magnesium in Figure 6c, such that the calcium ion concentration decreases in both the anode and cathode phases. The calcium consumption rate also increases after the silica is removed from the solution. These data suggest that hydroxide ions produced at the cathode are preferentially consumed to create the silica-magnesium-calcium compound and only then are used to generate the calcium compound. Based on the ICP-AES and EDS analyses, the precipitate formation reaction in Equation (3) proposed by Sheikholeslami et al. [21] appears to be responsible for the formation of the relatively large product. However, the calcium compound (calcium carbonate) is generated by the following reaction:

Ca^2+^ + HCO_3_^−^ + OH^−^ → CaCO_3_↓ + H_2_O. (4)

Although this is the primary reaction, other reactions would also be expected to occur, and it is thought that the elemental composition shown in Table 2 was obtained as a result of all these reactions. 

Figure 6d shows that the sodium ion concentration increases in the cathode phase but decreases in the anode phase. The decrease in the anode phase is ascribed to the electrophoresis of sodium ions from the anode to the cathode phase. Sodium ions do not participate in the precipitate formation reactions since their ionization tendency is high, and so the sodium concentration is increased at the cathode side. Consequently, the precipitate did not contain much sodium and the sodium that was found in the product likely resulted from sodium ions incorporated during the drying process. 

**Figure 7:**
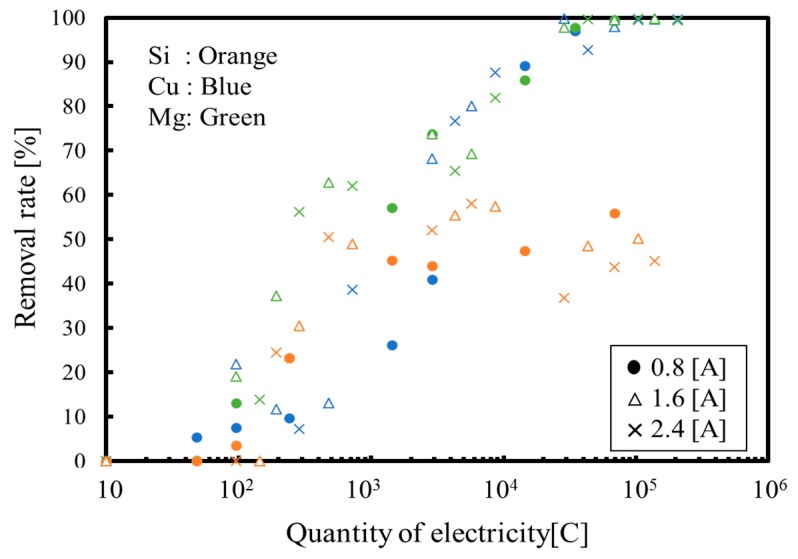
Removal rates as functions of the quantity of electricity.

Figure 7 shows the removal rate of each ion from both the anode and cathode phases, which can be considered as formation rate of each ion compound, since all ions removed from the solution are consumed in the precipitation formations. Note that the sodium ion that is not consumed in precipitation reactions is excluded from the figure. As can be seen from Figure 7, the removal rate of silica approaches 50 because the silica is not positively charged, and thus, cannot pass through the ion exchange membrane. In contrast, magnesium and calcium ions were completely removed from the solution. As shown in Figure 7, the removal rate of magnesium is fast when the silica precipitation reaction occurs, but on the other hand, after finishing the silica precipitation reaction, the calcium precipitation reaction increases. These data confirm that silica can be removed from the solution via a polymerization reaction with magnesium and calcium ions in solution, while calcium can be recovered as calcium carbonate following the formation of a silica-magnesium-calcium compound. However, these results are for a batch process, whereas a continuous process would be required for industrial applications.

### 3.2. Continuous Process

**Figure 8:**
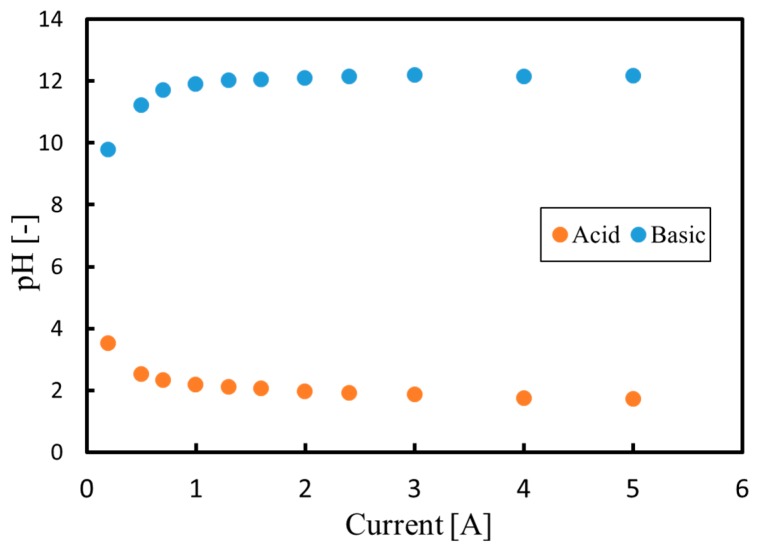
pH values during continuous processing as functions of current.

Figure 8 plots the pH values for both the anode and cathode phases at a flow rate of 50 mL/min as functions of the current in order to allow comparison with the batch test data in Figure 5. The pH increases in the cathode phase, but decreases in the anode phase, just as during the batch trials. Therefore, even during continuous processing, the same precipitate formation reactions as in the batch process can be expected. The formation of a white solid in the cathode phase was also observed during continuous processing. Furthermore, no channel clogging was evident, indicating that this process could be applied to continuous industrial operations.

**Figure 9:**
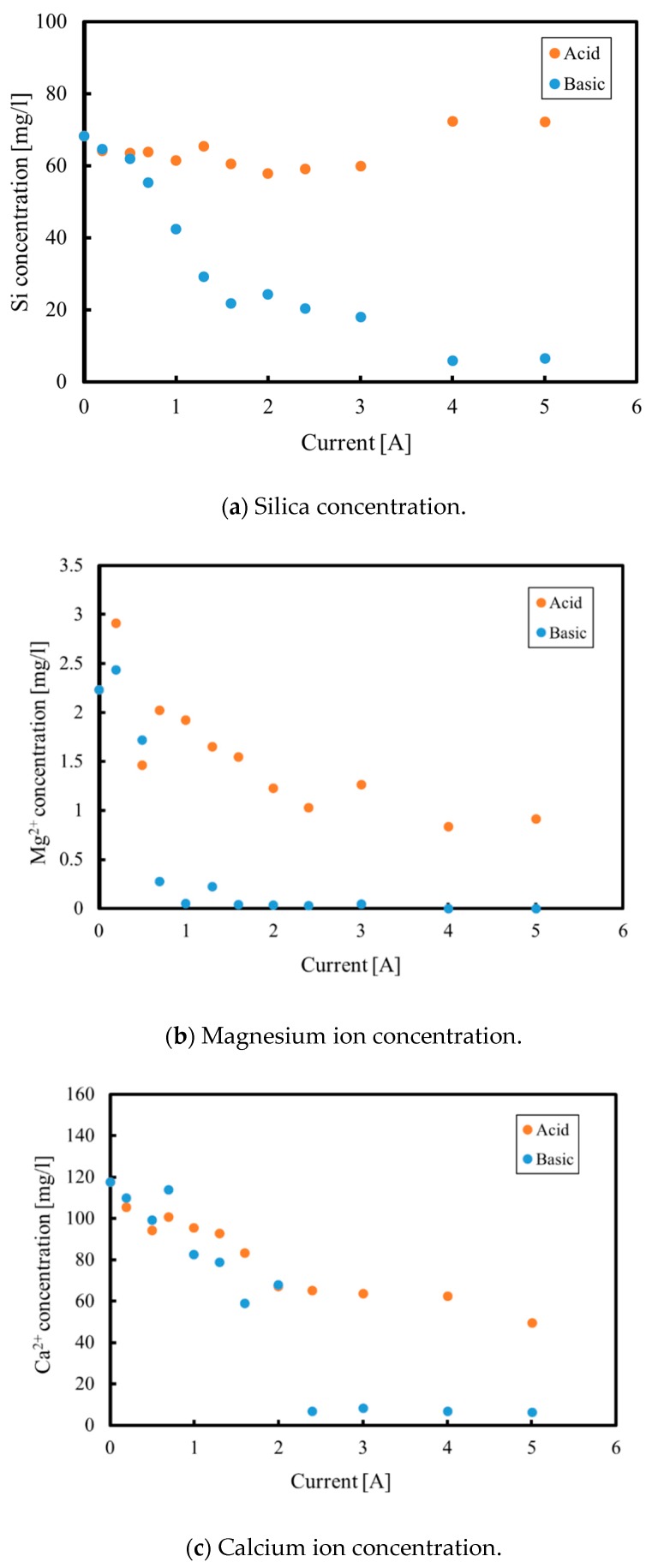

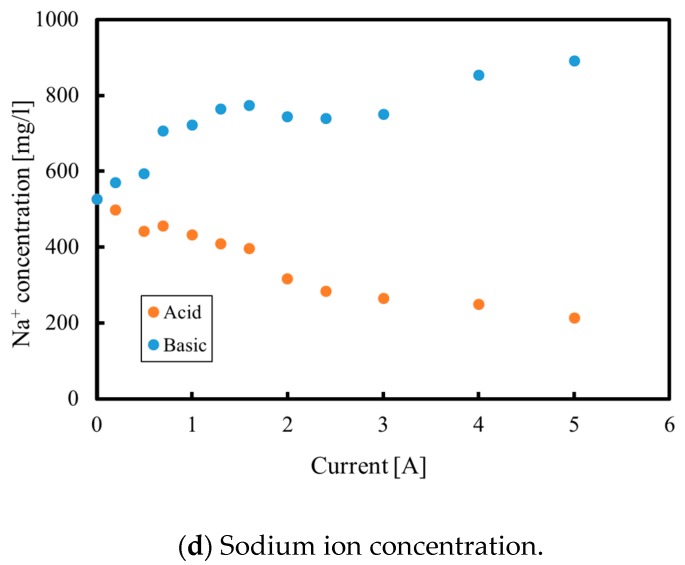
Concentrations of various elements as functions of the current during continuous processing. (**a**) Silica concentration; (**b**) Magnesium ion concentration; (**c**) Calcium ion concentration; (**d**) Sodium ion concentration.

Figure 9a–d shows the elemental concentrations in the electrolyzed solution during continuous processing. All elements exhibit the same trends that were observed during the batch tests. That is, the silica concentration decreases in the cathode phase but is constant in the anode phase, while magnesium and calcium concentrations decrease in both the anode and cathode phases. Conversely, the sodium concentration increases in the cathode phase while decreasing in the anode phase. The composition of the solid recovered from continuous processing was also similar to that generated during the batch experiments. However, Figure 9a demonstrates that a small amount of silica remained in the solution when applying a current of 5 A. The magnesium concentration in the Katase hot spring water used in the continuous experiments was lower than that in the Atagawa water used in the batch experiments, so it is possible that there was insufficient magnesium to remove all the silica in the cathode phase. Furthermore, under the over current of 3 A, the amounts of magnesium and calcium ions moving from the anode to the cathode decrease, because the relative concentrations of these ions are reduced due to the increase in the proton concentration. Therefore, the movement of other ions is inhibited when the proton concentration becomes high, meaning that the migration of protons to the cathode phase inhibits the precipitate formation reaction due to neutralization reactions.

**Figure 10:**
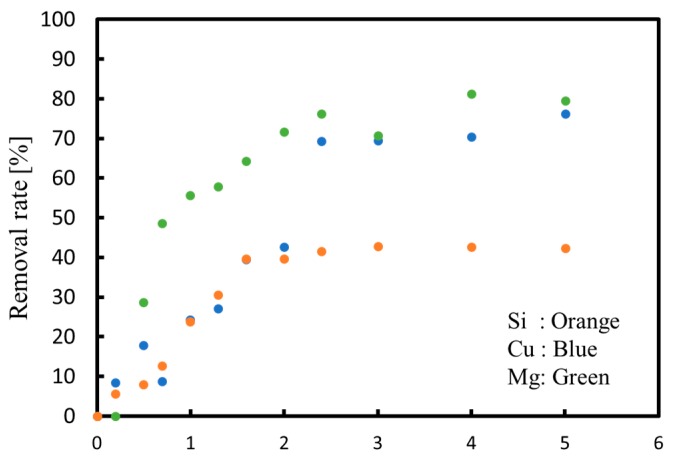
Removal rates as functions of the quantity of electricity during continuous processing.

Figure 10 shows the removal rate of each ion from both the anode and cathode phases. As explained above, it is clear that the magnesium concentration was not sufficient enough to remove all silica in the cathode phase, so that the removal rate of silica does not reach 50 in the figure. Furthermore, as can be seen from Figure 10, magnesium and calcium ions were not completely removed from the solution because the increase in the protons passing through to the cathode from the anode phase prevented magnesium and calcium immigrations.

The extent of the neutralization reactions due to protons passing from the anode to the cathode phase was assessed so as to examine the practical applicability of this method. In the proposed technique, when a current flows through the test device, ions pass through the cation exchange membrane from the anode to the cathode phase. Assuming that ion diffusion in the opposite direction is negligible and the all applied current is consumed for electrolysis, we can write the following equation:

(5) $$F\sum_{j}z_{j}J_{j}^{+}=I.$$

Here, *F* and *Z* are the Faraday constant and the valence of the ion, respectively, while *J_j_^+^* is the molar flow rate for each ion (mol/s) through the membrane from the anode to the cathode phase, and the subscript *j* denotes the ion (I = 1, 2, 3, 4 and 5 corresponds to Na^+^, Ca^2+^, K^+^, Mg^2+^ and H^+^, respectively). Assuming that these five ions carry the majority of the electric current, *I*, the mass balance equation obtained from Equation (5) is as follows:

(6) $$z_{5}J_{5}^{+}=\frac{I}{F}-\left(z_{1}J_{1}^{+}+z_{2}J_{2}^{+}+z_{3}J_{3}^{+}+z_{4}J_{4}^{+}\right).$$

The molar flow rates for ion numbers 1 to 4 can then be obtained from the ion concentrations in solution on the anode side using the equation below:

(7) $$J^{+}=q\left(c_{Acid,in}-c_{Acid,out}\right).$$

Here, *q* is the flow rate (m^3^/s) and the subscripts in and out denote the inlet and outlet of the test section. Both *I* and *J^+^_I_*_= 1–4_ can be measured, so the proton molar flow rate, *J^+^_I_*_= 5_, can be obtained from Equation (6). 

**Figure 11:**
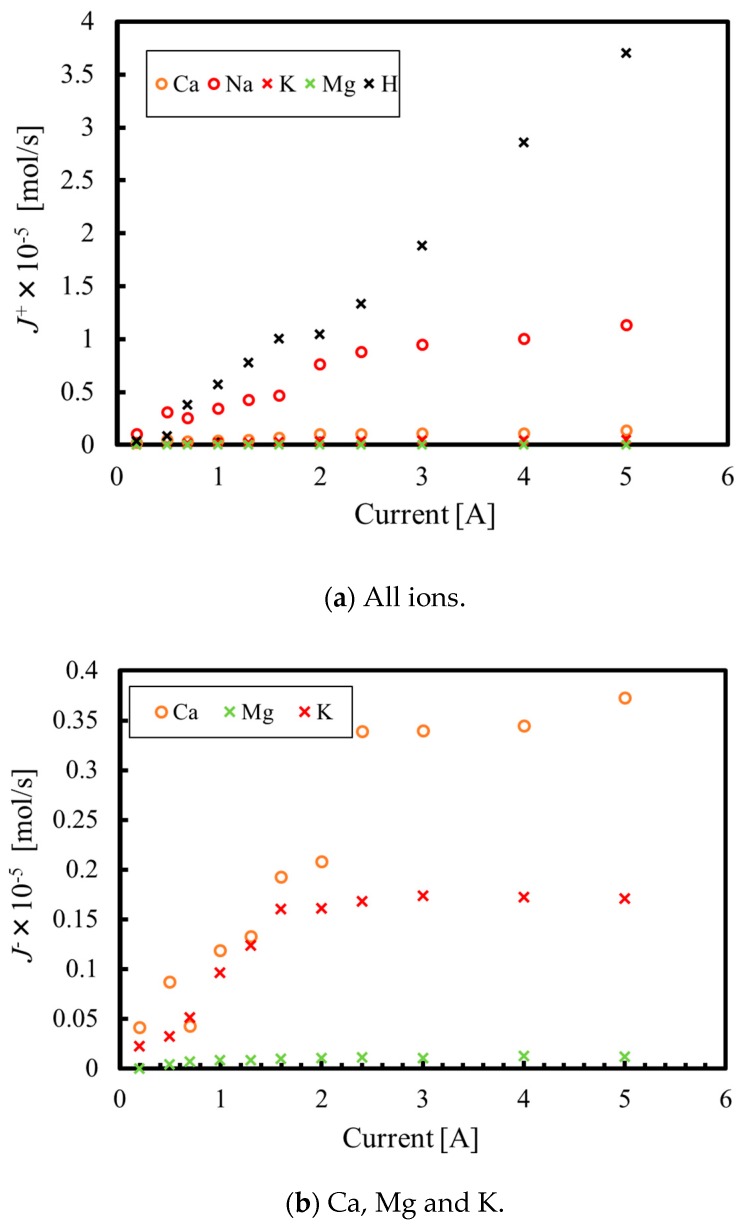
Molar flow rates for various ions as functions of the current. (**a**) All ions; (**b**) Ca, Mg and K.

Figure 11a plots the molar flow rates for all ions at a solution flow rate of 50 mL/min, while Figure 11b presents data for the low-concentration ions. At low currents, electricity is carried by the ions contained in the original hot spring water, since the proton concentration is low. In this region of the dataset, the molar flow rate through the membrane is correlated with the ion concentration in the solution, such that the molar flow rate increases in the order of Mg^2+^, K^+^, Ca^2+^ and Na^+^. The transportation of magnesium, potassium and calcium ions from the anode to the cathode phase is unaffected by changing the current between 0.6, 1.8 and 2.6 A, while the molar flow rate for the protons increases. In fact, protons carry about three times as much electricity as sodium ions at a current of 5 A. Neutralization reactions occur due to protons passing to the cathode from the anode phase. Therefore, at high current values, a part of the energy provided to the electrolysis system is wasted by the neutralization reactions. Protons become the main carrier of the current at 2.6 A, at which point all calcium ions in the cathode phase have been consumed by the formation of the precipitate. Thus, it is inefficient to apply the proposed method to prevent the generation of calcium carbonate scale. However, silica can be removed from the solution at low current values, so this method is considered to be effective at decreasing the silica concentration from the solution in geothermal binary power generation. Assuming that the original solution contains the magnesium or calcium ions that are required for polymerization reactions with silica, the silica concentration will be decreased to the desired concentration. Furthermore, if the both solutions for the anode and cathode that are passed through the proposed system are mixed and neutralized, it is possible to operate geothermal binary power generation with less environmental impact. To put this proposed system into practical use, a feasibility study and/or optimization of the device and conditions have to be necessary in the next steps. However, through this research, it was proven that the proposed method is an effective means of removing silica from a solution in geothermal binary power generation.

## 4. Conclusions

The viability of reducing the formation of calcium carbonate and silica scales by the reaction of silica and calcium with cations and hydroxide ions generated by water electrolysis was examined. Batch and continuous experiments demonstrated that all the silica in the cathode phase could be extracted in the presence of sufficient concentrations of magnesium and calcium. This was the result of the formation of a silica-magnesium-calcium compound. ICP-AES and EDS data showed that the composition of this compound was 2CaO-5MgO-8SiO_2_-H_2_O, which is in agreement with a prior report by Sheikholeslami et al. [21]. Calcium carbonate was subsequently formed. Channel blockage was not observed during the continuous operation of this process. Although protons supplied to the cathode phase inhibited the scale formation reaction, this effect should be mitigated to allow the efficient removal of calcium. The technique demonstrated herein appears to be an effective means of removing silica from a solution in geothermal binary power generation.

## Figures and Tables

**Table 1:** Specifications for the CMB membrane.

Electric resistance	Ω·cm^2^	4.5
Burst strength	Mpa	≥0.40
Thickness	Mm	0.21
Recommend Temperature	°C	≤60
Recommend pH	-	0–14

**Table 2:** Data from the elemental analyses of the precipitates by an energy dispersive X-ray spectroscopy (EDS).

Element	Molar Fraction (%)	
	5 min Later	24 h Later
Oxygen	68.58	72.05
Sodium	0.41	0.24
Magnesium	9.51	1.31
Silica	11.93	1.27
Chlorine	0.24	-
Calcium	9.06	25.13
Manganese	0.27	-
